# Foodborne Origin and Local and Global Spread of *Staphylococcus saprophyticus* Causing Human Urinary Tract Infections 

**DOI:** 10.3201/eid2703.200852

**Published:** 2021-03

**Authors:** Opeyemi U. Lawal, Maria J. Fraqueza, Ons Bouchami, Peder Worning, Mette D. Bartels, Maria L. Gonçalves, Paulo Paixão, Elsa Gonçalves, Cristina Toscano, Joanna Empel, Małgorzata Urbaś, M. Angeles Domínguez, Henrik Westh, Hermínia de Lencastre, Maria Miragaia

**Affiliations:** Universidade Nova de Lisboa, Oeiras, Portugal (O.U. Lawal, O. Bouchami, H. de Lencastre, M. Miragaia);; Centre for Interdisciplinary Research in Animal Health (CIISA), Universidade de Lisboa, Lisbon, Portugal (M.J., Fraqueza);; Hvidovre University Hospital, Hvidovre, Denmark (P. Worning, M.D. Bartels, H. Westh);; SAMS Hospital, Lisbon (M.L. Gonçalves);; Hospital da Luz, Lisbon (P. Paixão);; Hospital Egas Moniz, Lisbon (E. Gonçalves, C. Toscano);; Narodowy Instytut Leków, Warsaw, Poland (J. Empel, M. Urbaś);; Hospital Universitari de Bellvitge, Barcelona, Spain (M.A. Domínguez);; University of Copenhagen, Copenhagen, Denmark (H. Westh);; The Rockefeller University, New York, New York, USA (H. de Lencastre)

**Keywords:** urinary tract infections, UTIs, population structure, clone, origin, livestock, virulence, food, whole-genome sequencing, WGS, food safety, One Health, antimicrobial resistance, bacteria, MRSA and other staphylococci, zoonoses

## Abstract

*Staphylococcus saprophyticus* is a primary cause of community-acquired urinary tract infections (UTIs) in young women. *S. saprophyticus* colonizes humans and animals but basic features of its molecular epidemiology are undetermined. We conducted a phylogenomic analysis of 321 *S. saprophyticus* isolates collected from human UTIs worldwide during 1997–2017 and 232 isolates from human UTIs and the pig-processing chain in a confined region during 2016–2017. We found epidemiologic and genomic evidence that the meat-production chain is a major source of *S. saprophyticus* causing human UTIs; human microbiota is another possible origin. Pathogenic *S. saprophyticus* belonged to 2 lineages with distinctive genetic features that are globally and locally disseminated. Pangenome-wide approaches identified a strong association between pathogenicity and antimicrobial resistance, phages, platelet binding proteins, and an increased recombination rate. Our study provides insight into the origin, transmission, and population structure of pathogenic *S. saprophyticus* and identifies putative new virulence factors.

*Staphylococcus saprophyticus* is the cause of uncomplicated urinary tract infection (UTI) in 10%–20% of young women (*1*). Despite a greater successful treatment rate, *S. saprophyticus* UTI has a higher recurrent infection frequency than *Escherichia coli* UTI (*2*). Rare complications of *S. saprophyticus* UTI include acute pyelonephritis, nephrolithiasis, and endocarditis (*3*).

*S. saprophyticus* frequently colonizes humans and can be found in the gastrointestinal tract, vagina, and perineum (*4*). *S. saprophyticus* also is part of the gut and rectal flora of livestock, including pigs and cattle, and a frequent contaminant of meat and fermented food products (*1*). *S. saprophyticus* also has been recovered from polluted aquatic environments (*5*).

The reservoirs of *S. saprophyticus* causing UTI in humans are believed to be endogenous, but evidence is lacking. Moreover, given the frequent bacterial contamination of meat through the meat-processing chain, meat and food are speculated to be sources of human gut colonization and human *S. saprophyticus* infection (*6*). Some studies have reported high genetic diversity among isolates from human infections, food products, and other sources (*5*,*7*,*8*). However, previous studies were performed with a limited number of isolates, which prevented the description of the global and local molecular epidemiology of *S. saprophyticus*.

We used phenotypic, genomic, and pangenome-wide association study (pan-GWAS) approaches to characterize *S. saprophyticus* both globally and locally. In addition, we identified adaptive features that drive *S. saprophyticus* evolution, defined the *S. saprophyticus* population structure, investigated dissemination routes, and identified new pathogenicity factors.

## Methods

### Ethics Considerations

 The human isolates used in our study were recovered as part of routine clinical diagnostic testing; thus, ethics approval and informed consent were not required. All data were handled anonymously. Sample collection was in accordance with the European Parliament and Council decision for the epidemiologic surveillance and control of communicable disease through the European Antimicrobial Resistance Surveillance Network (https://www.ecdc.europa.eu/en/activities/surveillance/EARS-Net/Pages/index.aspx). Slaughterhouse samples were part of the routine control practices for evaluation of good hygiene practices and programs to assure meat safety (European Parliament and Council regulation no. 853/2004).

### Bacterial Isolates

The global *S. saprophyticus* collection we used included 299 isolates from humans collected in 7 countries during 1997–2017: 286 from UTIs, 12 from invasive disease, and 1 from colonization (Appendix 1 Table 1). We also analyzed the genomes of *S. saprophyticus* for 38 isolates from 5 other countries: 35 isolates from human UTIs (*8*), 2 from human hand swabs (*8*), an isolate from Byzantine Troy (*8*), and ATCC 15305 (*9*), a previously investigated human UTI-causing isolate. 

The local collection included isolates collected in Lisbon, Portugal, during 2016–2017: 128 human UTI isolates collected in 3 hospitals and 104 slaughterhouse isolates collected from equipment, pork samples, workers’ hands, and a pig’s rectum. In addition, we included 5 isolates from animals and 12 isolates from food used in other studies (*8*) (Appendix 2).

### Whole-Genome Sequencing and Assembly 

#### Phylogenetic Analysis

**Figure 1:**
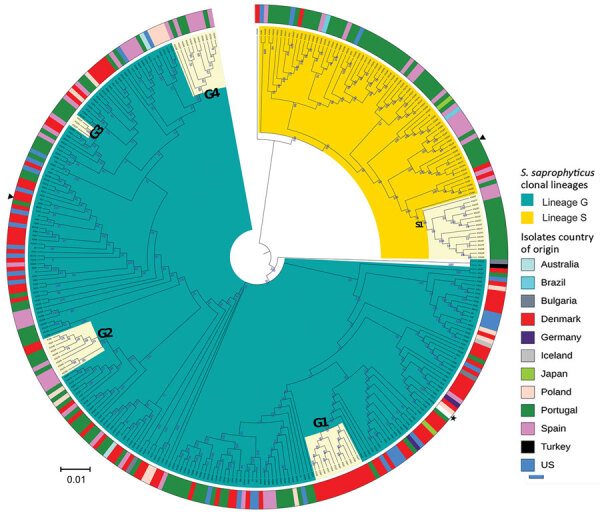
Maximum-likelihood tree of *Staphylococcus saprophyticus* isolates recovered from human infections and colonization globally, 1997–2017. The tree was constructed by using 9,134 SNPs without recombination. Among analyzed isolates, 321 were recovered from UTIs, 12 from blood, and 4 from colonization. Each node represents a strain; nodes with identical color belong to the same lineage. The assembled contigs were mapped to the reference genome *S. saprophyticus* ATCC 15305 (GenBank accession no. AP008934.1; black star). Polymorphic sites resulting from recombination events in the single-nucleotide polymorphism (SNP) alignments were filtered out by using Gubbins version 2.3.4 (*12*). Maximum likelihood tree was reconstructed by using RAxML version 8.2.4 (https://github.com/stamatak/standard-RAxML). We performed generalized time-reversible nucleotide substitution model with gamma correction with 100 bootstraps random resampling for support. We visualized the tree by using Interactive Tree of Life (iTOL; https://itol.embl.de). Black triangles represent isolates fully sequenced by using the long-read nanopore technologies and used as reference to estimate r/m in the respective lineage. Cream color represents clusters G1, G2, G3, G4, and S1, which had dissemination and transmission in same country and in different countries. The outer ring represents isolates’ country of origin; blocks with identical color represent isolates from the same country. Of note, cluster G4 contains a pair of isolates collected in 2016 that had only 10 SNPs difference; one is a blood isolate from Barcelona, Spain (KS266) and the other is a UTI isolate recovered in Lisbon, Portugal (KS135). Scale bar indicates number of substitutions per site. UTI, urinary tract infection; r/m, recombination to mutation ratio.

**Figure 3:**
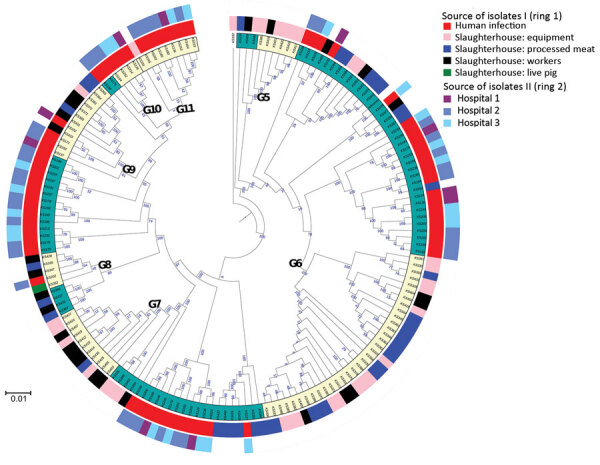
Maximum-likelihood tree depicting the genetic relatedness of *Staphylococcus saprophyticus* isolates belonging to clonal lineage G recovered from human infections or slaughterhouse contamination, Portugal, 2016–2017. Each node represents a strain. The tree was visualized by using Interactive Tree of Life (iTOL; https://itol.embl.de). Clusters highlighted in cream in the innermost ring represent admixture of isolates in clusters G5–G11, which were recovered from different sources and are closely related by single-nucleotide polymorphism. Ring 1 represents genetic relatedness of isolates recovered from different sites in the slaughterhouses and those recovered from infection in the community. Ring 2 depicts the isolates recovered from different hospitals. Scale bar indicates nucleotide substitutions per site.

We performed whole-genome sequencing (WGS) on MiSeq (Illumina, https://www.illumina.com) and MinIon nanopore (Oxford Nanopore, https://nanoporetech.com) platforms, as described (*10*) (Appendix 2). We separately analyzed global population and local epidemiology of *S. saprophyticus* and their phylogeny by using single-nucleotide polymorphisms (SNPs). We identified SNPs by mapping the draft genomes to a reference genome, *S. saprophyticus* ATCC 15305 (GenBank accession no. AP008934.1) by using the web-based CSI phylogeny version 1.4 (*11*) with the default parameter, but we disabled the minimum distance between SNPs in the parameter. We used Gubbins version 2.3.4 (*12*) with default parameters to concatenate SNPs and remove recombination regions. We reconstructed the phylogenies by using RAxML version 8.2.4 (https://github.com/stamatak/standard-RAxML) and generalized time-reversible nucleotide substitution with gamma correction model with 100 bootstrap value. We visualized the maximum-likelihood trees by using Interactive Tree of Life (ihttps://itol.embl.de) (Figures 1–3; Appendix 2 Figure 3). Recombination to mutation (r/m) ratios detected by using Gubbins were calculated as the average r/m of isolates in the entire collection and separately for each lineage by using as reference closed genomes of KS40 for lineage G and KS160 for lineage S, both obtained on the MinIon platform (Oxford Nanopore).

#### Pan-GWAS 

We used Prokka version 1.13 (https://vicbioinformatics.com/software.prokka.shtml) to annotate genomes and defined the pangenome by using 85% blastp (https://blast.ncbi.nlm.nih.gov/Blast.cgi) identity in Roary version 3.12 (http://sanger-pathogens.github.io/Roary). We performed GWAS by using Scoary version 1.6.16 (*13*) to identify genes associated with lineages and considered Bonferroni corrected p<0.05 (odds ratio [OR] >1) statistically significant; we identified genes associated with epidemiologic groups and considered Benjamini Hochberg corrected and pairwise comparison p<0.05 statistically significant. Sequence data from this study are available in GenBank (accession no. PRJNA604222).

#### Resistome and Virulome Analyses

 We screened genomes for resistance and virulence genes by using ResFinder version 2.3 (https://cge.cbs.dtu.dk/services/ResFinder), an in-house virulence genes database, and the virulence factor database (*14*), integrated into ABRicate version 0.5 (https://github.com/tseemann/abricate). We considered genes with a threshold >90% nucleotide identity and >60% coverage to be present.

### Statistical Analyses

We used Prism 6.0 (GraphPad, https://www.graphpad.com) to compare the means of 2 groups. We used a 2-tailed unpaired Mann-Whitney test or χ^2^ test for comparison and considered p<0.05 statistically significant.

## Results

### Lineages of *S. saprophyticus* Causing UTIs 

Among study isolates, we first analyzed the diversity of *S. saprophyticus* causing UTIs by using genomic data of 321 human UTI isolates collected from 8 countries on 4 continents during 1997–2017. From the SNPs initially detected, 42% arose from recombination events in the population, corresponding to a mean genome-wide r/m of 1.5:1, meaning that high accumulation of SNPs was due to recombination rather than mutation in UTI strains. The maximum-likelihood tree constructed from the 9,134 SNPs without recombination defined 2 lineages, which we called G and S (Figure 1). Most (74%, 236/321) UTI isolates were from lineage G and differed by 0–4,318 SNPs with an average nucleotide identity (ANI) of 98.5%–99.999%, whereas S isolates (26%, n = 85/321) were slightly less distantly related, differing by 0–3,540 SNPs with an ANI of 99.3%–99.991% (Appendix 2 Figure 1).

**Figure 4:**
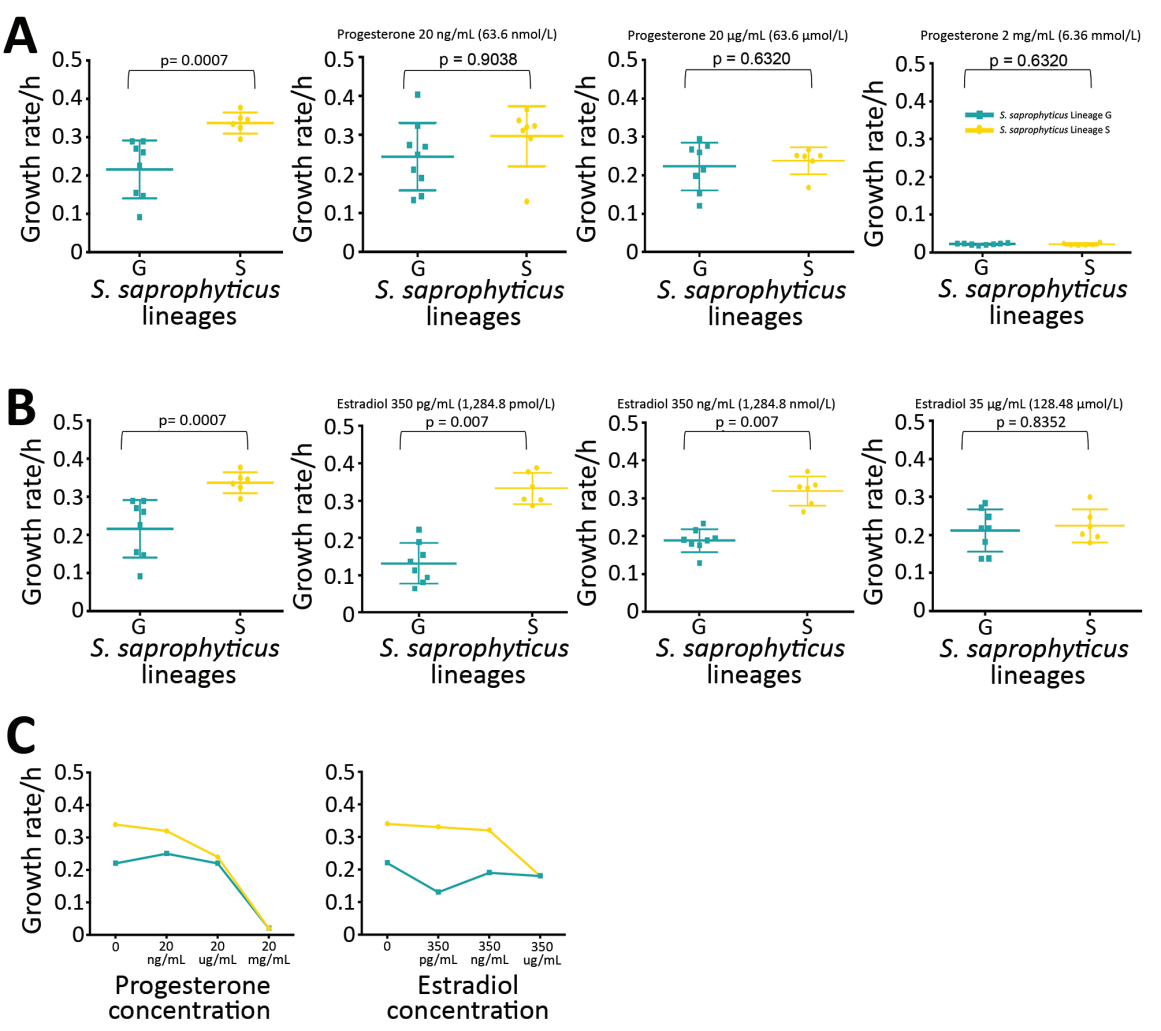
Growth rate of *Staphylococcus saprophyticus* clonal lineages in tryptic soy broth (TSB) and in different concentrations of female sex hormones. All assays were performed in triplicate and each experiment was repeated 3 times. A) Growth rate of *S. saprophyticus* strains in different concentrations of progesterone. First panel represents growth rate in TSB at 37°C; isolates belonging to lineage S grew significantly faster (p = 0.0007) than isolates in lineage G in TSB without hormones. However, no statistically significant difference in the growth rate of either lineage was noted in physiologic (2.0–200 ng/mL) and higher concentrations of progesterone. B) Growth rate of *S. saprophyticus* strains in TSB (first panel) and different concentrations of estradiol. Lineage S isolates grew faster in physiologic concentrations (350 pg/mL–350 ng/mL) and higher of estradiol, suggesting that this lineage is better adapted to the hormone-rich environment of the urine and the vagina than lineage G. Error bars indicate 95% CIs; horizontal lines indicate medians. C) Growth rate mean values of *S. saprophyticus* strains in progesterone and estradiol.

*S. saprophyticus* lineages we identified among UTI isolates worldwide had distinctive features indicative of 2 evolutionary histories. Although strains of both lineages had similar genome size as determined by their closed genomes (lineage G was 2.5 Mb and S 2.6 Mb), isolates from lineage S (n = 6) grew significantly faster in tryptic soy broth incubated at 37°C (μ_average_ = 0.34 h^–1^) than G strains (n = 8) (μ_average_ = 0.22 h^–1^; p = 0.0007) (Figure 4; Appendix 2). Moreover, we separately analyzed r/m of both lineages by using the respective closed genome of strains from each lineage as a reference and found that the mean estimated r/m was 9 times higher in S isolates (r/m = 4.4:1) than G isolates (r/m = 0.5:1). We did not detect a temporal signal when performing regression analysis (r) of tip-to-root distance versus isolation date, either in the entire collection (r = −0.2423) or for separate lineages (for G, r = −0.1314; for S, r = 0.1889) (Appendix). Hence, we could not determine when the 2 lineages diverged. The lack of temporal signal probably results from the limited number of isolates in each time point.

**Figure 2:**
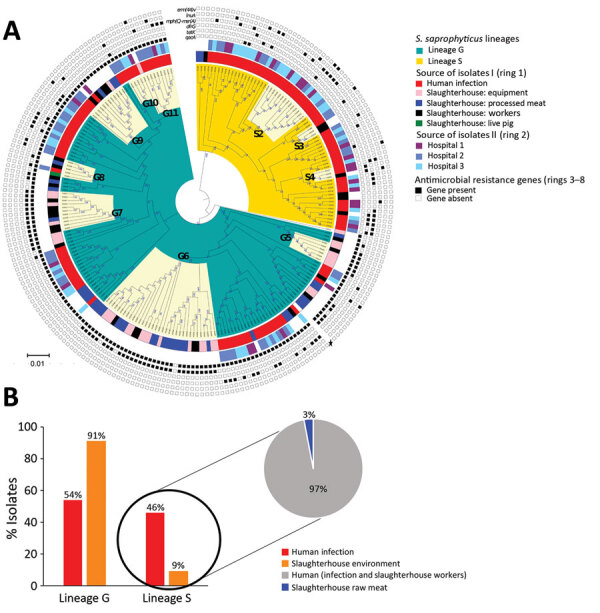
Phylogenomic analysis and distribution of *Staphylococcus saprophyticus* isolates collected from human infections and a slaughterhouse, Portugal, 2016–2017. A) Maximum-likelihood tree of 232 isolates from human infections or slaughterhouse contamination. The tree was constructed by using 14,110 single-nucleotide polymorphisms (SNPs) without recombination. Each node represents a strain; nodes with identical color belong to the same lineage. The assembled contigs were mapped to the reference genome *S. saprophyticus* ATCC 15305 (GenBank accession no. AP008934.1; black star). SNPs generated from each genome were concatenated to single alignment corresponding to position of the reference genome. Polymorphic sites resulting from recombination events in the SNP alignments were filtered out by using out by using Gubbins version 2.3.4 (*12*). Tree was reconstructed by using RAxML version 8.2.4 (https://github.com/stamatak/standard-RAxML). The generalized time-reversible nucleotide substitution model with gamma correction was performed with 100 bootstrap random re-samplings for support. The tree was visualized by using Interactive Tree of Life (iTOL; https://itol.embl.de). The clusters highlighted in cream represent admixture of isolates recovered from different sources that are closely related by SNPs in clusters G5–G11 and S2–S4. The inner ring (ring 1) represents genetic relatedness of isolates recovered from different sites inside the slaughterhouses and those recovered from infection in the community. The center ring (ring 2) identifies the isolates recovered from different hospitals. The outer rings (rings 3–8) represent the distribution of 6 genes that convey antimicrobial resistance. Scale bar indicates nucleotide substitutions per site. B) Source-based distribution of *S. saprophyticus* isolates in the lineage G and lineage S. Lineage G consisted isolates from infections, colonization, and contamination. Almost all (97%) isolates in lineage S are from human colonization and infection.

**Figure 5:**
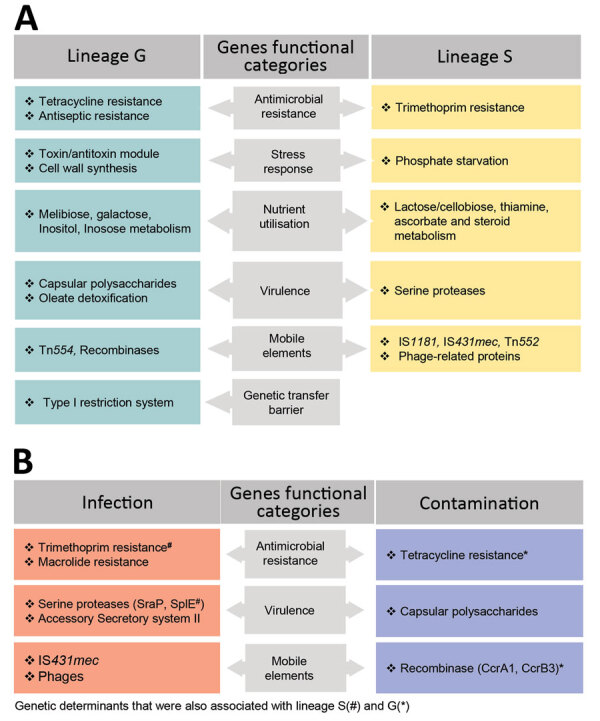
Genetic determinants that contribute to the distinction of clonal lineages and lifestyle of *Staphylococcus saprophyticus*. The graph displays determinants that contribute (A) and mediate (B) adaptation of *S. saprophyticus* to either infection or contamination. We used the genome-wide association study (GWAS) method to identify genetic factors by using 2 association comparisons: lineage G versus lineage S and human infection versus surface contamination. We used the pairwise comparison and included a core-SNP phylogenetic tree without recombination to remove the lineage effect in the analysis. Hits with Benjamini Hochberg corrected p<0.05 and odds ratio >1 were considered statistically significant. We grouped the identified genes into biologic functions based on gene annotation predicted by Prokka (https://vicbioinformatics.com/software.prokka.shtml). Some genetic factors that were associated with infections and contamination also were associated with the lineages despite subjecting the GWAS to lineage correction.

To further compare the 2 lineages, we constructed the pangenome of the 338 human *S. saprophyticus* genomes and identified 10,222 genes with 85% blastp clustering by using Roary. Among these, 8,351 genes, present in <99% of the genomes, constituted the accessory genome. A gene accumulation plot of all the genes against the genomes sequenced showed that *S. saprophyticus* has an open pangenome (Appendix 2 Figure 2, panels A, B). The pan-GWAS analysis of the accessory genome indicated that G and S isolates have different genomic content (Bonferroni p<0.05, OR >1). A total of 128 genes were specific/enriched in the G lineage, including those encoding a type I restriction subunit (*group_383*), a defense mechanism against genetic transfer (*15*); metabolism of melibiose (*ebgA*, *melB*) (*16*) and inositol (*iolE*) (*17*), compounds that are excreted in urine; toxin–antitoxin systems (*group_4685*, *group_5665*), involved in stress response (*18*); and antimicrobial resistance (*qacA*) (*19*) (Table 1; Figure 5, panel A; Appendix 2 Table 1).

**Table 1:** List of genes exclusively associated with lineage G *Staphylococcus saprophyticus* strains in study of foodborne origin and local and global spread of *S. saprophyticus* causing human urinary tract infections*

Genes	Gene predicted function	Biologic function group	Frequency, %	Reference no.
*group_383*	Type I site-specific deoxyribonuclease restriction subunit	Endonuclease	96	(*15*)
*icaR*	Ica operon HTH-type negative transcription regulator	Transcription	95	NA
*licT*	Transcription antiterminator LicT	Transcription	80	NA
*iolE*	Inosose dehydratase	Inositol metabolism	86	(*17*)
*group_4976*	Phage infection protein	Phage-related protein	46	NA
*group_869*	Bacteriophage integrase	Phage-related protein	42	NA
*tnpC_1*	Transposase for transposon Tn*554*	Mobile genetic element	17	NA
*group_744*	Putative glucarate transporter	Transporter	29	NA
*group_4685*	Addiction module toxin Txe/YoeB family protein	Stress response (Type II toxin–antitoxin system)	20	(*18*)
*group_5665*	Addiction module antitoxin Axe family protein	Stress response (Type II toxin–antitoxin system)	19	(*18*)
*yhjQ*	Putative cysteine-rich protein YhjQ	Putative function	17	NA

*List of genes also include 53 hypothetical proteins. Bonferroni adjusted p<0.032. NA, not applicable.

For S lineage, 237 genes were specific/enriched, some of which are involved in metabolism of ascorbate (*ulaA*) and thiamine (*tenI*) (*20*); induction of phosphate starvation (*psiE*), which was previously linked to switch on virulence in other uropathogens (*21*); platelet binding (*splE*, *sdrE*) associated with virulence (*22*); steroid metabolism (*group_7190*); and resistance to trimethoprim (*dfrG*) and biocides (*qacC*) (Table 2; Figure 5, panel A; Appendix 2 Table 1). Additional genes associated with S lineage that could explain its increased growth rate include those linked to lactose and cellobiose metabolism (*group_5572* gene) (*23*) and cell wall hydrolysis (*lytN*) (*24*), whereas the prevalence of transposases (*group_3547* and *group_1828*) (*25*) and phage genes (*recT* and *yueB*) (*27*) could justify its increased recombination rate.

**Table 2:** List of lineage S genes exclusively associated with *Staphylococcus saprophyticus* strains in study of foodborne origin and local and global spread of human *S. saprophyticus* urinary tract infections *

Genes	Gene predicted function	Biologic function group	Frequency, %	Reference no.
*group_5572*	Phosphotransferase system, lactose/cellobiose-specific IIB subunit	Sugar metabolism	99	(*23*)
*ulaA*	PTS system ascorbate-specific IIC component	Sugar metabolism	99	(*20*)
*group_5955*	Sugar phosphate isomerase/epimerase	Sugar metabolism	26	(*23*)
*lytN*	C51 family D-Ala-D-Gly carboxypeptidase	Cell wall hydrolase	85	(*24*)
*psiE*	Protein PsiE	Phosphate starvation	22	(*21*)
*group_1438*	Arsenite methyltransferase	Arsenite resistance	25	NA
*dfrG*	Trimethoprim-resistance dihydrofolate reductase	Antimicrobial resistance	18	NA
*group_7190*	3-β hydroxysteroid dehydrogenase/isomerase	Steroid metabolism	17	(*26*)
*group_273*	Recombinase/resolvase	Mobile genetic element	69	(*25*)
*group_2198*	Putative ATPase/transposase	Mobile genetic element	26	(*25*)
*group_275*	Recombinase/resolvase	Mobile genetic element	21	(*25*)
*group_355*	Transposase for IS*431mec*	Mobile genetic element	17	(*25*)
*group_7470*	Putative replication-associated protein	Mobile genetic element	10	﻿NA
*group_3363*	putative DoxX family membrane protein	Putative functions	85	NA
*mviM*	NADH-dependent dehydrogenase	Putative functions	26	NA
*nmrA*	Putative nmrA negative transcriptional regulator family protein	Transcription	17	NA
*group_7195*	Amidohydrolase	Hydrolase	17	NA
*group_6430*	Putative restriction enzyme	Restriction enzyme	17	NA

*List of genes also include 84 hypothetical proteins. Bonferroni p<0.031. NA, not applicable.

### Local and Global Spread of *S. saprophyticus* Clones Causing UTIs 

We observed no time-based clustering of *S. saprophyticus* UTI isolates in the phylogeny, but we noted some geographic clustering. In particular, 89% (n = 78) of S isolates were found in Portugal and Spain (Figure 1). In addition, we identified clusters containing isolates from a single country. For instance, cluster G3 (1–8 SNPs) contained only isolates from Portugal and cluster G1 (0–63 SNPs) only isolates from Denmark (Figure 1).

We noted a high degree of isolate admixture in the maximum-likelihood tree of our global collection, suggesting that *S. saprophyticus* isolates of both lineages are disseminated geographically. G strains were distributed most widely, in 11 countries on 4 continents, whereas we found S isolates in only 6 countries. Despite the genetic diversity described, we still found isolates from different countries that differed by only a few SNPs. One pair in cluster G4 that had only 10 SNPs difference was a blood isolate from Barcelona, Spain (KS266), and a UTI isolate from Lisbon (KS135), collected in 2016 (Figure 1).

Although a relatedness cutoff is not defined yet for *S. saprophyticus*, the low number of SNPs observed between strains from the same and different countries is below the relatedness cutoff of 10–40 SNPs for most bacterial species (*28*). The apparent relatedness we noted implies that UTI isolates from different patients in the same country and in different countries are highly related and could belong to a cross-border chain of transmission. This finding challenges the assumption that *S. saprophyticus* causing UTIs were mainly endogenous (*29*).

### Genetic Relatedness of *S. saprophyticus* from Slaughterhouses and UTIs 

Pork is the most frequently consumed red meat in Europe (*30*) and is often contaminated with *S. saprophyticus* (*1*). We found that 35% of slaughterhouse samples (from meat, equipment, workers’ hands, and a live pig) were contaminated with *S. saprophyticus*. 

To understand whether *S. saprophyticus* causing UTIs could be related to *S. saprophyticus* in pork, we compared 104 isolates collected from a slaughterhouse against 128 isolates collected from human UTIs in Lisbon during 2016–2017. Among the 104 isolates from the slaughterhouse, 39 (37.5%) were collected from meat, 32 (30.8%) from equipment, 32 (30.8%) from workers’ hands, and 1 (≈1%) from a live pig. SNP-based phylogenetic analysis with a tree-rooted at the midpoint showed that most (91%; 95/104) slaughterhouse isolates belonged to lineage G (Figure 2, panels A, B) and that a strain from slaughterhouse equipment was at the base of this lineage (bootstrap 100). In addition, the phylogenetic reconstruction including isolates from this study and other isolates from production and companion animals (including 2 pigs, 2 bovine, and 1 canine) and food (*8*) showed that most (3/5) animal isolates clustered together at a basal clade of lineage G (bootstrap 100) (Appendix 2 Figure 2). Some clusters in the phylogenetic tree (e.g., G9) had slaughterhouse isolates at the base and UTI isolates at the tip. However, we also observed the opposite (e.g., G11), tree clusters with slaughterhouse isolates at the tip and UTI isolates at the base (Figure 2, panel A). Moreover, 41% of G strains included the antimicrobial resistance gene *tetK* (p<0.0001) (Figure 2, panel A), which is associated with resistance to tetracycline, an antimicrobial drug extensively used in animal production (*31*).

Phylogenetic reconstruction of all isolates from Lisbon based on SNPs provided additional examples of admixture of isolates recovered from different sampling sites in the slaughterhouse and from the slaughterhouse and humans. Isolates from meat were frequently intermixed with isolates from equipment and colonized workers as observed in cluster G6, wherein stains differed by only 1–65 SNPs (Figure 2, panel A; Figure 3). Likewise, cluster G9 included isolates from the slaughterhouse that were intermixed with human UTI isolates. Isolates collected from slaughterhouse equipment differed by only 19 SNPs from human UTI isolates. Likewise, human UTI isolates differed from meat isolates by 25 SNPs and from isolates of colonized workers by 26 SNPs. In addition, slaughterhouse and human UTI isolates had the same antimicrobial resistance profile, exhibiting resistance to fosfomycin, fusidic acid, and tetracycline (Appendix 1 Table 1). We could not ascertain any epidemiologic link between the workers in the slaughterhouse and UTI patients, due to data protection limitations, but contact with meat production previously has been identified as a risk factor for UTI (*6*).

The only live-pig isolate was intermixed in cluster G8 with isolates from meat, slaughterhouse workers, and UTI patients (Figure 3), having 307–658 SNPs difference. The admixture of strains in the tree suggests the existence of frequent cross-transmission within the slaughterhouse and between the slaughterhouse and humans.

Insufficient disinfection procedures probably contributed to the high transmission rate of *S. saprophyticus* within the slaughterhouse, as demonstrated by the highly related strains (<11 SNPs) on dirty and clean equipment surfaces and similar strains (<16 SNPs) isolated 12 months apart. Carriage of the antimicrobial resistance gene, *qacA*, by all G strains could justify the observed unsuccessful cleaning procedures (Figure 2, panel A).

The accumulation of substitutions and genetic distance evidenced by the phylogenetic analysis suggest that isolates from slaughterhouses and food probably are the primary sources of *S. saprophyticus* G strains (Figure 3). Transmission probably occurs more frequently from the slaughterhouse and food to humans; however, we cannot ascertain directionality due to the lack of temporal signal.

### Evidence Supporting the Human Origin of Lineage S

In contrast to isolates belonging to lineage G, which were mostly from UTIs and the slaughterhouse, S isolates were almost exclusively of human origin (97%; n = 66/68), either from UTIs (n = 59) or human colonization (n = 7) (Figure 2, panels A, B). When we reconstructed the phylogeny of all isolates in this and other studies (*8*) (Appendix 2 Figure 3), a human isolate was at the base of the S lineage. The only 2 S isolates seen in animals were from nonhuman primates. Furthermore, a resistance determinant for trimethoprim (*dfrG*), which routinely is used to treat human UTIs, was associated with this lineage (18%; p<0.05) (Figure 2, panel A; Table 2).

To determine whether S isolates could have originated in humans, we grew isolates from both lineages in the absence and presence of human physiologic concentrations (15–350 pg/mL) of estradiol (*32*), a female hormone commonly found in urine and the vagina, and in different pH values mimicking the stomach (pH 2.5), vagina (pH <4.5) (*33*), skin (pH 5.5), and urine (pH 4.5–8.0) (*34*). The growth rate of S isolates did not change significantly at the highest physiologic concentration of estradiol specific to humans (0.34 h^–1^ vs. 0.33 h^–1^), but the growth rate for G strains decreased by 59% at this concentration (0.22 h^–1^ vs. 0.13 h^–1^; p = 0.0007) (Figure 4, panels A–C; Appendix 2). All isolates grew at all pH levels assayed, except for pH 2.5. At pH 4.5 and 5.5, isolates of both lineages had similar growth rates, but S isolates had a higher growth rate than G isolates at pH 8, although this difference was not statistically significant (p = 0.133). These results suggest that lineage S isolates are more adapted than lineage G strains to high estradiol concentrations found in women, but not found in other female animal hosts, such as pigs, bovine, or canines (*35*). GWAS also identified a gene involved in steroid metabolism, 3-β hydroxysteroid dehydrogenase (HSD), associated with lineage S (Table 2; Figure 5, panels A, B). Steroids such as estradiol are primary signaling molecules for host–microbe interactions (*26*) and involved in the interconversion of active and inactive steroid hormones (*26*). Presence of HSD could be an adaptive evolution to colonization of the bladder, a hormone-rich environment. Evidence supports a human (primate) origin for lineage S, but studies sampling a wider range of ecologic sites and geographic regions are needed.

### UTIs and *S. saprophyticus* Dissemination among Humans in the Community

To explore dissemination of *S. saprophyticus* causing UTIs in the community through human-to-human contact, we analyzed genomic data of the 128 UTI isolates from outpatients of 3 hospitals in the Lisbon area. Transmission of *S. saprophyticus* from lineage G and S between persons in the community was apparent, as demonstrated by the high relatedness of strains from UTI patients at different hospitals. In the G cluster, G10 isolates had 9–24 SNPs difference, and in the S cluster, S2 isolates differed by 6–64 SNPs (Figure 2, panel A). However, due to data protection regulations, we could not ascertain whether the patients were epidemiologically linked. Results suggest that patients might have acquired these strains from the same reservoir or that direct and indirect cross-transmission could have occurred in the community.

### Disease Signatures among *S. saprophyticus* Populations

Several virulence factors, including urease, have been described in *S. saprophyticus* (*36*), but the basis of pathogenicity in this species is mainly unknown. We used a pan-GWAS approach to compare the genetic content of 128 isolates from human infections to 104 isolates recovered from a slaughterhouse, all collected in Lisbon during 2016–2017. We identified 6 genes that appear to be associated with an increased pathogenic potential in *S. saprophyticus* (Figure 5, panel A; Appendix 2 Tables 2–5). These genes included those encoding an SplE-like protein and a gene cluster encoding a complete accessory secretory system associated with a serine-rich adhesion, similar to SraP. Previous studies have described highly homologous secretory system (>97% nucleotide identity) associated to serine-rich proteins that bind platelets, including SraP in *S. aureus* (*37*) and UafB in *S. saprophyticus* (*38*).

We also found other genes associated with infection that encoded phage proteins. An analysis on 128 UTI isolates using PHASTER (*39*) identified 5 phages in 48 (38%) strains. The phages were 38–125 Kb and had <50% identity with any known phage. The genomic vicinity of the identified phages varied in the chromosome, suggesting that lysogenic conversion was not the mechanism involved in pathogenicity of these strains; instead, hypothetical phage genes could be crucial for pathogenicity. In addition, genes encoding resistance to the antimicrobial drugs trimethoprim (*dfrG*), lincosamide (*lnuA*), streptogramin B (*erm44v*), or to macrolides (*mphC-msrA*) also were associated with strains from human infections (Figure 2; Figure 5, panel B; Appendix 2 Tables 2–5).

Another factor highly associated with human infections was the occurrence of recombination, which was 6 times higher in isolates from UTIs (r/m 1.7:1) than from isolates associated with colonization or contamination (r/m 0.3:1). This finding suggests recombination might be a strategy of *S. saprophyticus* to evade the host immune system, as described elsewhere (*40*).

## Discussion

We identified 2 *S. saprophyticus* lineages, G and S, associated with human UTIs that appeared to have different evolutionary histories. Our data support a foodborne origin for lineage G and its transmission through food products to humans. We also found evidence of a human origin for lineage S and additional proof for the occurrence of direct or indirect human-to-human dissemination in the community, which could explain not only the local dissemination of both lineages but also the wide geographic dissemination, as described for *S. aureus* (*41*) and *E. coli* (*42*).

Our conclusions were limited by the characteristics of the sample collection analyzed. In particular, the lack of temporal signal did not enable inference of the direction of transmission between the sampling sites. We could establish obvious genomic relatedness between the meat production chain and human UTIs and UTIs from different persons in the community. However, lack of samples from other animal hosts, environments, or different ecologic niches in humans, did not enable us to establish pig meat and humans as the unique sources of the 2 lineages nor identify the preferred ecologic niche of *S. saprophyticus* in humans.

**Figure 6:**
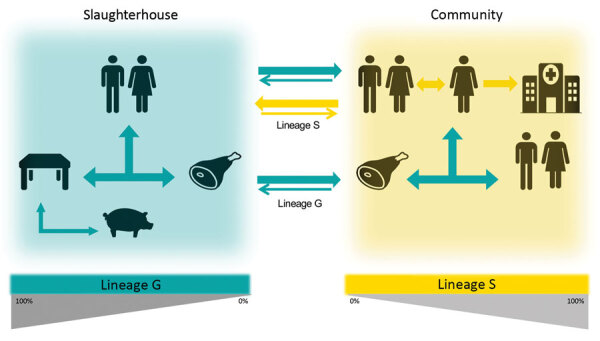
A proposed model for the dissemination and transmission of *Staphylococcus saprophyticus* in the community. The arrows represent the dissemination and transmission of *S. saprophyticus* isolates that belonged to lineage G (green) and lineage S (yellow). Lineage G *S. saprophyticus* strains are of animal origin and enter the slaughterhouse through production animals, such as pigs, persist on the equipment, and contaminate the meat in processing chain. Lineage G strains could enter the community through contaminated meat and workers colonized in the slaughterhouse. Lineage S strains most likely are of humans and primate origin and probably are disseminated by person-to-person contact within the community.

Our observed *S. saprophyticus* colonization rate among pigs was extremely low (1%) compared with previous studies (*43*), which could be explained by possible cross-inhibitory bacterial interactions (*44*); however, contamination within the slaughterhouse environment sometimes reached 35%. Amplification of *S. saprophyticus* in the slaughterhouse environment was probably potentiated by resistance to biocides. We found that slaughterhouse workers’ washed hands were colonized with strains that were highly related to slaughterhouse environmental isolates and to strains causing UTIs, suggesting exposure of workers to the slaughterhouse environment as a risk factor for human colonization (Figure 6).

The transmission chain of S lineage isolates appears to be different and to have no link to meat production. S lineage UTIs might originate from human gut or vaginal colonization; both have been reported as possible human niches (*2*,*4*). Evidence for the human origin of S lineage included the almost exclusive (97%) identification in humans, where S lineage was better adapted to the physiologic high concentration of human female sex hormones; grew at vagina, skin, and urine pH values; and harbored 2 serine-proteases that presumably can bind to human platelets, as described for SraP (*37*). The distinct genetic content and phenotypic features of lineages G and S further reflected the diverse selective pressures of the human and animal, slaughterhouse, and food environments and suggest different evolutionary strategies toward pathogenicity. In particular, we found that tetracycline and antimicrobial resistance genes associated with colonization-contamination also were associated with isolates of the G lineage. Furthermore, genes encoding sugar metabolism, serine proteases, and a trimethoprim resistance associated with infection were also associated with isolates of the S lineage, implying distinctive specialization of the 2 lineages.

Our results also indicate a key role of setting-associated antimicrobial drug usage, especially for trimethoprim, macrolides, and tetracycline, in resistance development and pathogenicity. Subinhibitory concentrations of these drugs have been shown to promote virulence in bacteria through the induction of biofilm production (*45*), quorum-sensing (*45*), or phages (*46*) and might also increase *S. saprophyticus* pathogenicity.

We found a high r/m rate in isolates of the S lineage and in strains from UTIs, comparable to naturally transformable bacterial species like *Klebsiella pneumoniae* and *Streptococcus pyogenes* (*47*). A similar phenomenon was observed for *S. epidermidis* (*48*); variation in cell surface proteins was shown to contribute to evasion of human immunity (*48*), and a similar strategy might be advantageous for *S. saprophyticus* in infection. The recombination might have resulted from defects in repair of double stranded DNA breaks from oxidative stress induced by leukocytes during infection, as previously described (*48*). However, the mechanism linking infection and recombination in *S. saprophyticus* remains elusive.

Last, we identified factors associated with infection that could represent new *S. saprophyticus* virulence factors, including 2 serine-proteases, *sraP*-like and *splE*-like, and phages SraP and SplE, which previously were connected to pathogenicity in *S. aureus* (*22*,*37*). In addition, phages have been described to transport pathogenicity islands in staphylococci (*49*). Our study constitutes a deep-structured analysis of *S. saprophyticus* population structure and genomic epidemiology, providing groundwork for future studies on the pathogenicity and population genetics of this bacterium.

Appendix 1 Information on strains and phylogenetic relatedness of *Staphylococcus saprophyticus* in study of isolates from human urinary tract infections and meat processing plants.

Appendix 2 Additional laboratory methods and information on *Staphylococcus saprophyticus* in a study of isolates from human urinary tract infections and meat processing plants.
