# Supplementary material for: Foodborne Origin and Local and Global Spread of Staphylococcus saprophyticus Causing Human Urinary Tract Infections
Source: Emerg Infect Dis. 2021 Mar;27(3):880–93. doi: 10.3201/eid2703.200852 (PMC7920669; doi:10.3201/eid2703.200852)
Supplement: Appendix 2 — Additional laboratory methods and information on Staphylococcus saprophyticus in a study of isolates from human urinary tract infections and meat processing plants. [file 20-0852-Techapp-s2.pdf]

Article DOI: https://doi.org/10.3201/eid2703.200852

Foodborne Origin and Local and Global Spread of *Staphylococcus saprophyticus* Causing Human Urinary Tract Infections

Appendix 2

Supplementary Methods

Bacterial Isolates

In addition to the global and local *Staphylococcus saprophyticus* collections stated in the manuscript, we included *S. saprophyticus* isolates from food production animals (1 pig and 3 bovine), 1 companion canine, 12 food isolates, and 1 isolate recovered from a river, which was described in previous studies (*1*). We also included 18 *S. saprophyticus* isolates recovered from food products and 2 isolates from nonhuman primates from our collection. These isolates, together with the global and local collection (n = 480) was analyzed to infer the origin of the *S. saprophyticus* lineages.

Isolation and Species Identification of Slaughterhouse Isolates

We enriched samples in peptone water and grew isolates on CHROMagar Staph aureus (CHROMagar Microbiology, https://www.chromagar.com) supplemented with 10% NaCl and 4 µg/mL novobiocin. We extracted genomic DNA by using methods previously described (*2*). We performed species identification by amplifying and sequencing the *tuf* gene (*3*).

Growth Rate in Different pH and Hormones

For representative isolates from the collection, we performed growth curves at different concentrations of female sex hormones, including progesterone at 20 ng/mL, 20 µg/mL, and 20 mg/mL, and estradiol at 350 pg/mL, 350 ng/mL, and 350 µg/mL; and at pH levels of 2.5, 4.5, 5.5, and 8.0. We performed growth assays by using an Infinite 200 PRO series microtiter reader (Tecan Group Ltd, https://www.tecan.com) in 96-well microtiter plates. For each strain, overnight culture was inoculated onto 5 mL Bacto tryptic soy broth (TSB; Becton Dickinson,

https://www.bd.com). The OD_{600nm} of the liquid culture was adjusted to an initial OD of 0.5 MacFarland with buffers for specific pHs and TSB containing varying concentrations of hormones and grown with aeration (180 rpm) at 37°C for 18 h. Assays were performed in triplicate and each experiment was repeated 3 times.

Estimation of Evolutionary Rates

To estimate the evolutionary rates in *S. saprophyticus* population, as a first approach, we explored the degree and pattern of temporal signal and determined whether sufficient temporal signals were available in the *S. saprophyticus* phylogeny. We performed a regression of the divergence of each tip from the root against the date of sampling, a root-to-tip plot, of the global collection and separately for the lineages using TempEst v1.5.3 (*4*). We used the phylogenetic tree without recombination and the date of isolation of the isolates as inputs.

Average Nucleotide Identity Analysis

We calculated average nucleotide identity (ANI) for representative strains of *S. saprophyticus* 40 G lineages and 20 S lineages by using a standalone Python program, pyani version 0.2.9 (https://github.com/widdowquinn/pyani) and the ANIb option, which compares genomes using BLAST program (https://blast.ncbi.nlm.nih.gov). The closed genome of KS40 was used as a reference for lineage G and closed genome of KS160 was used for lineage S.

Intrasample Diversity

We assessed the genetic diversity between isolates recovered from the same sample in the meat processing chain. We determined whether intrasample diversity existed by comparing the SNP differences between these isolates.

Data Availability

All raw sequence data are available in the SRA (https://www.ncbi.nlm.nih.gov/sra) under the study accession no. PRJNA604222. We also provide individual accession numbers for raw sequence data (Appendix 1 Table 1) and the SNP matrices and list of genes in the pangenomes (Appendix 1 Tables 2–6).

Results

Pangenome Analysis of *S. saprophyticus* Revealed an Open Pangenome

We annotated the 338 *S. saprophyticus* genomes by using Prokka (*5*) and constructed the pangenome by using Roary (*6*) with 85% blastp identity. A total of 10,222 genes were found, 48% (n = 4,925) of which were genes with unknown functions. The genes constituting the core of all isolates consisted of 1,871 genes. Also, we noted 118 soft core genes in 95%–99% of the isolates, 856 shell genes in 15%–94%, and we found 7,307 genes that constituted cloud genes in <15% of *S. saprophyticus* population. On average, 75% of *S. saprophyticus* genome is constituted by core genes and 25% of accessory genes. The plot of the total number of genes against the number of genomes indicate an open pangenome in which each genome sequence added several new genes. This finding implies that newly sequenced genomes will identify new genes and the pangenome size of this species will continue to increase (Appendix 2 Figure 2).

GWAS Revealed Genetic Factors Associated with *S. saprophyticus* Isolates from Different Genetic Lineages and Clinical Origins

We explored the pangenome gene presence to understand the difference in the genetic content of isolates from each of the genetic lineages defined by core SNPs. We used Scoary pipeline (*7*) and Bonferroni p<0.05 to identify genes that were exclusive or enriched in the *S. saprophyticus* genetic lineages. We categorized the hits into biologic function groups based on the annotations predicted by Prokka. For genes associated with different clinical origins (infection and colonization/contamination), we used Benjamini Hochberg and pairwise p<0.05 (Appendix 2 Tables 1–5).

References

1. Mortimer TD, Annis DS, O'Neill MB, Bohr LL, Smith TM, Poinar HN, et al. Adaptation in a fibronectin binding autolysin of *Staphylococcus saprophyticus*. MSphere. 2017;2:e00511–17. PubMed https://doi.org/10.1128/mSphere.00511-17

2. Couto I, Pereira S, Miragaia M, Sanches IS, de Lencastre H. Identification of clinical staphylococcal isolates from humans by internal transcribed spacer PCR. J Clin Microbiol. 2001;39:3099–103. PubMed https://doi.org/10.1128/JCM.39.9.3099-3103.2001

3. Martineau F, Picard FJ, Ménard C, Roy PH, Ouellette M, Bergeron MG. Development of a rapid PCR assay specific for *Staphylococcus saprophyticus* and application to direct detection from urine

samples. J Clin Microbial. 2000;38:3280–4. PubMed https://doi.org/10.1128/JCM.38.9.3280-3284.2000

4. Rambaut A, Lam TT, Max Carvalho L, Pybus OG. Exploring the temporal structure of heterochronous sequences using TempEst (formerly Path-O-Gen). Virus Evol. 2016;2:vew007. PubMed https://doi.org/10.1093/ve/vew007

5. Seemann T. Prokka: rapid prokaryotic genome annotation. Bioinformatics. 2014;30:2068–9. PubMed https://doi.org/10.1093/bioinformatics/btu153

6. Page AJ, Cummins CA, Hunt M, Wong VK, Reuter S, Holden MTG, et al. Roary: rapid large-scale prokaryote pan genome analysis. Bioinformatics. 2015;31:3691–3. PubMed https://doi.org/10.1093/bioinformatics/btv421

7. Brynildsrud O, Bohlin J, Scheffer L, Eldholm V. Erratum to: Rapid scoring of genes in microbial pan-genome-wide association studies with Scoary. Genome Biol. 2016;17:1–9. PubMed https://doi.org/10.1186/s13059-016-1108-8

8. Tomita K, Nagura T, Okuhara Y, Nakajima-Adachi H, Shigematsu N, Aritsuka T, et al. Dietary melibiose regulates the cell response and enhances the induction of oral tolerance. Biosci Biotechnol Biochem. 2007;71:2774–80. PubMed https://doi.org/10.1271/bbb.70372

9. Costliow ZA, Degnan PH. Thiamine acquisition strategies impact metabolism and competition in the gut microbe *Bacteroides thetaiotaomicron.* [Internet]. mSystems. 2017;2:1–17 http://msystems.asm.org/lookup/doi/10.1128/mSystems.00116-17. PubMed https://doi.org/10.1128/mSystems.00116-17

10. Sharp JA, Echague CG, Hair PS, Ward MD, Nyalwidhe JO, Geoghegan JA, et al. Staphylococcus aureus surface protein SdrE binds complement regulator factor H as an immune evasion tactic. PLoS One. 2012;7:e38407. PubMed https://doi.org/10.1371/journal.pone.0038407

11. Hallet B, Sherratt DJ. Transposition and site-specific recombination: adapting DNA cut-and-paste mechanisms to a variety of genetic rearrangements. FEMS Microbiol Rev. 1997;21:157–78. PubMed https://doi.org/10.1111/j.1574-6976.1997.tb00349.x

12. Datta S, Costantino N, Zhou X, Court DL. Identification and analysis of recombineering functions from Gram-negative and Gram-positive bacteria and their phages. Proc Natl Acad Sci U S A. 2008;105:1626–31. PubMed https://doi.org/10.1073/pnas.0709089105

13. Imperi F, Leoni L, Visca P. Antivirulence activity of azithromycin in *Pseudomonas aeruginosa.* Front Microbiol. 2014;5:178. PubMed https://doi.org/10.3389/fmicb.2014.00178

14. Goerke C, Köller J, Wolz C. Ciprofloxacin and trimethoprim cause phage induction and virulence modulation in *Staphylococcus aureus.* Antimicrob Agents Chemother. 2006;50:171–7. PubMed https://doi.org/10.1128/AAC.50.1.171-177.2006

15. Siboo IR, Chaffin DO, Rubens CE, Sullam PM. Characterization of the accessory *Sec* system of *Staphylococcus aureus.* J Bacteriol. 2008;190:6188–96. PubMed https://doi.org/10.1128/JB.00300-08

16. King NP, Beatson SA, Totsika M, Ulett GC, Alm RA, Manning PA, et al. UafB is a serine-rich repeat adhesin of *Staphylococcus saprophyticus* that mediates binding to fibronectin, fibrinogen and human uroepithelial cells. Microbiology (Reading). 2011;157:1161–75. PubMed https://doi.org/10.1099/mic.0.047639-0

17. Monk IR, Foster TJ. Genetic manipulation of Staphylococci-breaking through the barrier. Front Cell Infect Microbiol. 2012;2:49. PubMed https://doi.org/10.3389/fcimb.2012.00049

18. Chopra I, Roberts M. Tetracycline antibiotics: mode of action, applications, molecular biology, and epidemiology of bacterial resistance. Microbiol Mol Biol Rev. 2001;65:232–60. PubMed https://doi.org/10.1128/MMBR.65.2.232-260.2001

Appendix 2 Table 1. List of differentially enriched genes in *Staphylococcus saprophyticus* lineages in a study of isolates from human urinary tract infections and meat processing plants*

Gene	Gene predicted function	Biologic function group	Lineage G, %	Lineage S, %	Reference no.
melB	Melibiose carrier protein	Sugar transport and metabolism	98	26	(*8*)
ebgA	Evolved β-galactosidase subunit α	Sugar transport and metabolism	98	26	(*8*)
csxA	Exo-β-D-glucosaminidase	Sugar transport and metabolism	62	1	(*8*)
arsA	Arsenical pump-driving ATPase	Metal resistance	28	2	NA
arsD	Arsenical resistance operon transacting repressor ArsD	Metal resistance	28	2	NA
tenI	Thiazole tautomerase	Thiamine biosynthesis	22	100	(*9*)
splE	S1B family serine protease SplE	Virulence	15	100	(*10*)
sdrE	Serine-rich repeat-containing protein	Virulence	35	74	(*10*)
mhpC	Arylesterase	Hydrolase	26	60	NA
group_1205	Transcriptional regulator	Transcriptional regulator	81	1	NA
group_4356	Rho termination factor domain-containing protein	Transcriptional regulator	4	59	NA
qacA	Antiseptic resistance protein	Biocide resistance	100	5	NA
qacC	Quaternary ammonium compound-resistance protein QacC	Biocide resistance	35	87	NA
group_2160	Chaperone ATPase	Putative functions	16	84	NA
group_330	Spore coat protein	Putative functions	12	46	NA
bin3_2	Recombinase/resolvase	Mobile genetic element	32	3	NA
group_4660	Putative replication-associated protein	Mobile genetic element	28	1	NA
group_2182	Putative replication-associated protein	Mobile genetic element	54	97	NA
group_3547	IS*1181* transposase	Mobile genetic element	45	78	(*11*)
group_1828	Transposase-associated ATP/GTP binding protein	Mobile genetic element	15	59	(*11*)
group_1679	Transposase for transposon Tn552	Mobile genetic element	2	25	(*11*)
group_278	Recombinase/resolvase	Mobile genetic element	1	20	(*11*)
yueB	Phage infection protein	Phage-related protein	38	85	(*12*)
recT	Putative phage-related DNA recombination protein	Phage-related protein	15	59	(*12*)
group_2472	Phage N-acetlyglucosaminidase	Phage-related protein	15	59	(*12*)
group_2102	Phage protein	Phage-related protein	15	59	NA
group_2856	Phage protein	Phage-related protein	15	59	NA
group_2857	Phage protein	Phage-related protein	15	59	NA
group_3414	Phage protein	Phage-related protein	15	59	NA
group_1521	Phage tape measure protein	Phage-related protein	15	59	NA
group_2854	Putative phage DNA-packaging protein	Phage-related protein	15	59	NA
group_2855	Putative phage head-tail adaptor	Phage-related protein	15	59	NA
group_4784	Putative phage minor structural protein	Phage-related protein	15	59	NA
group_1829	PVL phage protein	Phage-related protein	15	59	NA
group_2103	Phage N-acetlyglucosaminidase	Phage-related protein	15	57	NA
group_2858	Phage tail protein	Phage-related protein	15	56	NA
group_1640	Phage portal protein, SPP1 family	Phage-related protein	15	56	NA
group_3412	Phage terminase, large subunit	Phage-related protein	15	56	NA
group_2860	Phage protein	Phage-related protein	14	56	NA
group_4359	Holin protein	Phage-related protein	14	56	NA
group_3894	Phage minor structural protein GP20	Phage-related protein	14	47	NA
group_3895	Phage minor head protein	Phage-related protein	13	47	NA
group_4788	Phage transcriptional regulator	Phage-related protein	7	46	NA
group_3800	Bacteriophage transcriptional regulator	Phage-related protein	5	24	NA
group_4678	Bacteriophage integrase	Phage-related protein	5	24	NA
group_6539	Bacteriophage terminase small subunit	Phage-related protein	2	43	NA

*Bonferroni $p \leq 0.00002$. Genes encoding hypothetical proteins enriched in lineage G = 55; genes encoding hypothetical proteins enriched in lineage S = 9. NA, not applicable.

Appendix 2 Table 2. List of genes that were exclusively associated with *Staphylococcus saprophyticus* isolates recovered from urinary tract infections*

Gene	Gene predicted function	Biologic function group	% Infection	Reference no.
group_1652	Putative DNA primase-phage associated	Phage-related protein	15	NA
group_2800	Putative phage leukocidin protein	Phage-related protein	8	NA
group_4438	Phage minor structural GP20	Phage-related protein	8	NA
group_1406	Hypothetical protein	Uncharacterized protein	15	NA
group_1405	Hypothetical protein	Uncharacterized protein	15	NA
group_4443	Hypothetical protein	Uncharacterized protein	10	NA
group_3431	Hypothetical protein	Uncharacterized protein	10	NA
group_863	Hypothetical protein	Uncharacterized protein	9	NA
group_4447	Hypothetical protein	Uncharacterized protein	9	NA
group_4446	Hypothetical protein	Uncharacterized protein	9	NA
group_4445	Hypothetical protein	Uncharacterized protein	9	NA
group_4439	Hypothetical protein	Uncharacterized protein	9	NA
group_3617	Hypothetical protein	Uncharacterized protein	7	NA
group_3616	Hypothetical protein	Uncharacterized protein	7	NA
group_1466	Hypothetical protein	Uncharacterized protein	7	NA

*Benjamini Hochberg $p \leq 0.02$. NA, not applicable.

Appendix 2 Table 3. List of genes that were enriched in *Staphylococcus saprophyticus* isolates recovered from urinary tract infections*

Gene	Gene predicted function	Biologic function group	% Infection	% Contamination	Reference no.
group_4400	Mph(C) macrolide 2' phosphotransferase	Antimicrobial resistance	25	2	(*13*,*14*)
dfrG	Dihydrofolate reductase	Antimicrobial resistance	9	1	(*14*)
csoR	Copper-sensing transcriptional repressor CsoR	Metal resistance	28	3	NA
cadX	Putative cadmium efflux system accessory protein	Metal resistance	44	13	NA
rep	Plasmid replication initiation protein	Mobile genetic element	18	3	NA
group_2868	Protein rlx	Mobile genetic element	17	2	NA
group_422	Transposase for IS*431mec*	Mobile genetic element	14	2	(*11*)
group_425	Transposase for IS*431mec*	Mobile genetic element	12	2	(*11*)
group_1094	Bacteriophage integrase	Phage-related protein	38	8	(*12*,*14*)
group_4449	DNA packaging protein Staph phage phiRS7	Phage-related protein	19	4	(*12*,*14*)
group_858	Holin protein	Phage-related protein	16	2	(*12*,*14*)
group_1653	Phage protein	Phage-related protein	15	2	(*14*)
group_1392	Phage protein	Phage-related protein	13	2	(*14*)
group_800	Bacteriophage tail tape measure protein	Phage-related protein	13	2	(*14*)
splE	S1B family serine protease SplE	Virulence	55	24	(*10*)
group_3377	Accessory Sec system protein Asp1	Virulence	27	3	(*15*,*16*)
secY_2	Preprotein translocase subunit SecY2	Virulence	27	3	(*15*,*16*)
secA2	Sec family Type I general secretory pathway protein SecA2	Virulence	27	3	(*15*,*16*)
asp3	Accessory Sec system protein Asp3	Virulence	27	3	(*15*,*16*)
asp2	Accessory Sec system protein Asp2	Virulence	27	3	(*15*,*16*)
sraP	Serine-rich repeat-containing protein	Virulence	14	1	(*15*,*16*)
grxC	Glutaredoxin 3	Stress response	30	7	NA
kefF	Glutathione-regulated potassium-efflux system ancillary protein KefF	Stress response	16	3	NA
yhjQ	Putative cysteine-rich protein YhjQ	Stress response	9	1	NA
splE	S1B family serine protease SplE	Virulence	55	24	(*10*)
group_3377	Accessory Sec system protein Asp1	Virulence	27	3	(*15*,*16*)

Gene	Gene predicted function	Biologic function group	% Infection	% Contamination	Reference no.
secY_2	Preprotein translocase subunit SecY2	Virulence	27	3	(*15*,*16*)
secA2	Sec family Type I general secretory pathway protein SecA2	Virulence	27	3	(*15*,*16*)
asp3	Accessory Sec system protein Asp3	Virulence	27	3	(*15*,*16*)
asp2	Accessory Sec system protein Asp2	Virulence	27	3	(*15*,*16*)
sraP	Serine-rich repeat-containing protein	Virulence	14	1	(*15*,*16*)
grxC	Glutaredoxin 3	Stress response	30	7	NA
kefF	Glutathione-regulated potassium-efflux system ancillary protein KefF	Stress response	16	3	NA
yhjQ	Putative cysteine-rich protein YhjQ	Stress response	9	1	NA

*Benjamini Hochberg p≤0.01. Hypothetical proteins (n = 19 genes). NA, not applicable.

Appendix 2 Table 4. List of genes that were exclusively associated with *Staphylococcus saprophyticus* recovered from environmental sources*

Gene	Gene predicted function	Biologic function group	% Contamination	References
group_1467	Hypothetical protein	Uncharacterized protein	42	NA
group_466	Hypothetical protein	Uncharacterized protein	39	NA
group_1991	Hypothetical protein	Uncharacterized protein	32	NA
group_6148	Hypothetical protein	Uncharacterized protein	18	NA
group_6146	Hypothetical protein	Uncharacterized protein	18	NA

*Benjamini Hochberg p≤0.00006. NA, not applicable.

Appendix 2 Table 5. List of genes that were enriched for in *Staphylococcus saprophyticus* isolates recovered from environmental sources*

Gene	Gene predicted function	Biologic function group	% Contamination	% Infection	References
group_227	Type I site-specific deoxyribonuclease restriction subunit	Restriction system	83	32	(*17*)
ccrB	Cassette chromosome recombinase B	Mobile genetic element	61	9	NA
ccrA	Cassette chromosome recombinase A1	Mobile genetic element	54	4	NA
group_1878	Putative replication-associated protein	Mobile genetic element	49	17	NA
group_3001	Myosin-cross reactive antigen (Oleate hydratase)	Stress tolerance	60	6	NA
tetK	Tetracycline resistance protein	Antimicrobial resistance	51	12	(*18*)
cap5O	Capsular polysaccharide biosynthesis protein Cap5O	Capsule	15	1	NA
cap5M	Capsular polysaccharide biosynthesis galactosyltransferase Cap5M	Capsule	13	2	NA
group_1472	Hypothetical protein	Uncharacterized protein	86	56	NA
group_1179	Hypothetical protein	Uncharacterized protein	69	24	NA
group_353	Hypothetical protein	Uncharacterized protein	61	9	NA
group_2224	Hypothetical protein	Uncharacterized protein	59	18	NA
group_1627	Hypothetical protein	Uncharacterized protein	55	25	NA
group_2291	Hypothetical protein	Uncharacterized protein	17	1	NA
group_3863	Hypothetical protein	Uncharacterized protein	10	1	NA

*Benjamini Hochberg p≤0.0001. NA, not applicable.

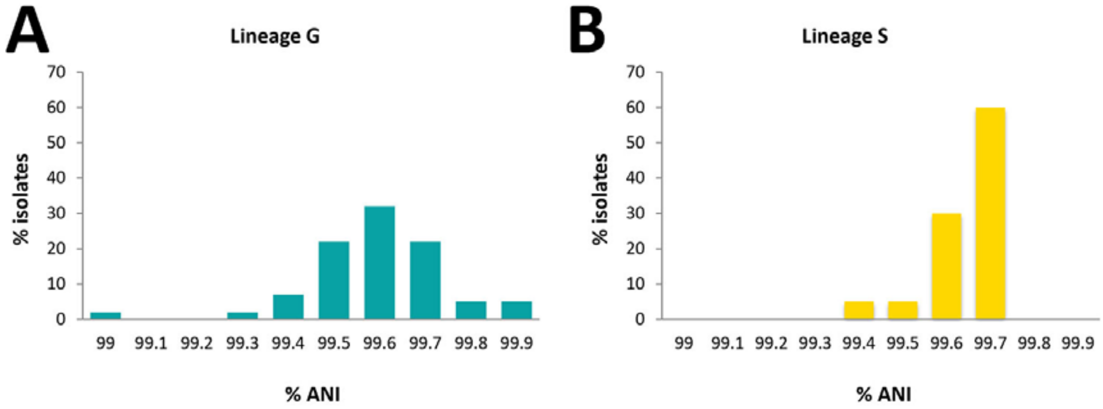

Appendix 2 Figure 1. Measure of genetic diversity between *Staphylococcus saprophyticus* lineage G (A) and lineage S (B) determined by using ANI. Lineage G strains had ANI values of 98.5%–99.999% and appear to be more diverse compared with lineage S strains, which had ANI values of 99.3%–99.991% and were slightly less diverse. Most isolates in lineage G had a lower ANI (99.6%) than the isolates in lineage S (99.7%). The ANI results were comparable to the genetic diversity observed with single nucleotide polymorphism analysis. ANI, average nucleotide identity.

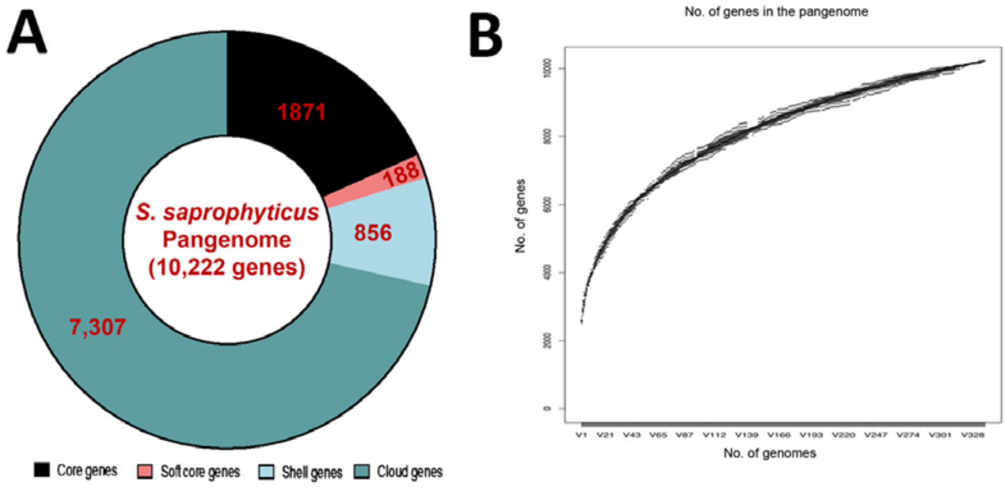

Appendix 2 Figure 2. Pangenome of *Staphylococcus saprophyticus* inferred from 338 isolates recovered from human infections and colonization. Among analyzed isolates, 321 were recovered from UTIs, 12 from blood, 4 from colonization, and 1 from reference strain ATCC 15305 (https://www.atcc.org; GenBank accession no. AP008934.1). A) Distribution of genes in the pangenome generated using Roary (*6*). We found a total of 10,222 genes. The core genes shared by all isolates were constituted of 1,871 genes. We also found 188 soft core genes in 95%–99% of isolates, and 856 shell genes in 15%–94% of isolates. In addition, we noted 7,307 cloud genes <15% of *S. saprophyticus* population. B) Gene accumulation plot for *S. saprophyticus* pangenome as a function of genomes sequenced indicating that *S. saprophyticus* has an open pangenome.

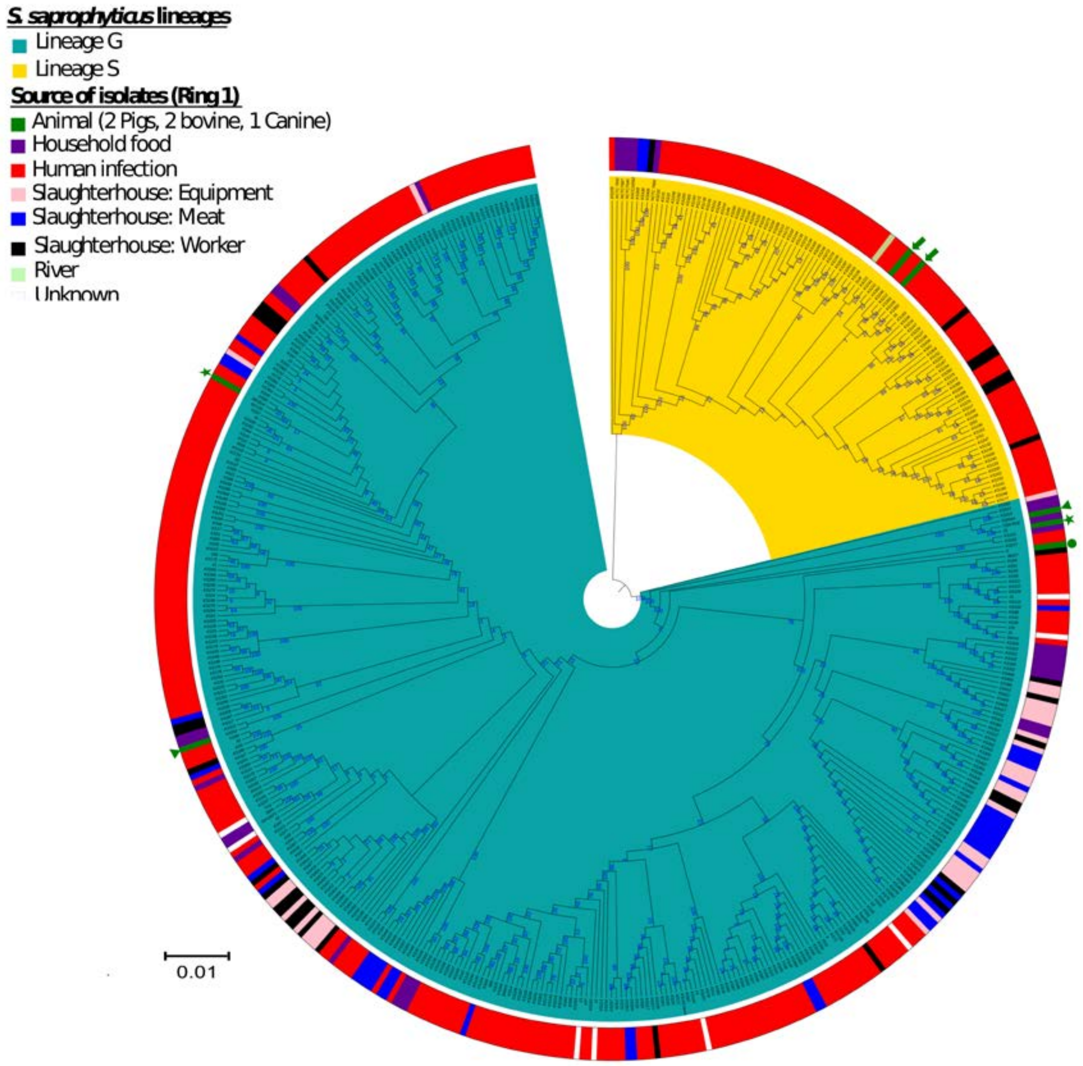

Appendix 2 Figure 3. Single nucleotide polymorphism-based maximum likelihood tree of 480 *Staphylococcus saprophyticus* from different sources. Each node represents a strain. A node with identical color belongs to the same lineage. The assembled contigs were mapped to the reference genome *S. saprophyticus* ATCC 15305 (https://www.atcc.org; GenBank accession no. AP008934.1) and SNPs were called. SNPs generated from each genome were concatenated to single alignment corresponding to position of the reference genome. Polymorphic sites resulting from recombination events in the SNP alignments were filtered out by using Gubbins v2.3.4 (Sanger, https://sanger-pathogens.github.io/gubbins). Maximum likelihood tree was reconstructed using RAxML version 8.2.4 (https://github.com/stamatak/standard-RAxML). The generalized time reversible nucleotide substitution with gamma correction was performed with 100 bootstraps random resampling for support. The image was generated using Interactive Tree of Life (https://itol.embl.de). The colored ring represents the source

of isolates. The green triangles represent strains recovered from pigs, green stars represent strains from bovines, and green circle a strain from a domestic canine. A slaughterhouse isolate recovered from equipment and those from pigs, bovines, and canine were at the base of lineage G, suggesting a probable foodborne origin of this lineage. Conversely, a human infection isolate was at the base of lineage S implying a human origin of this lineage. The green arrows in lineage S depict isolates recovered from small nonhuman primates.

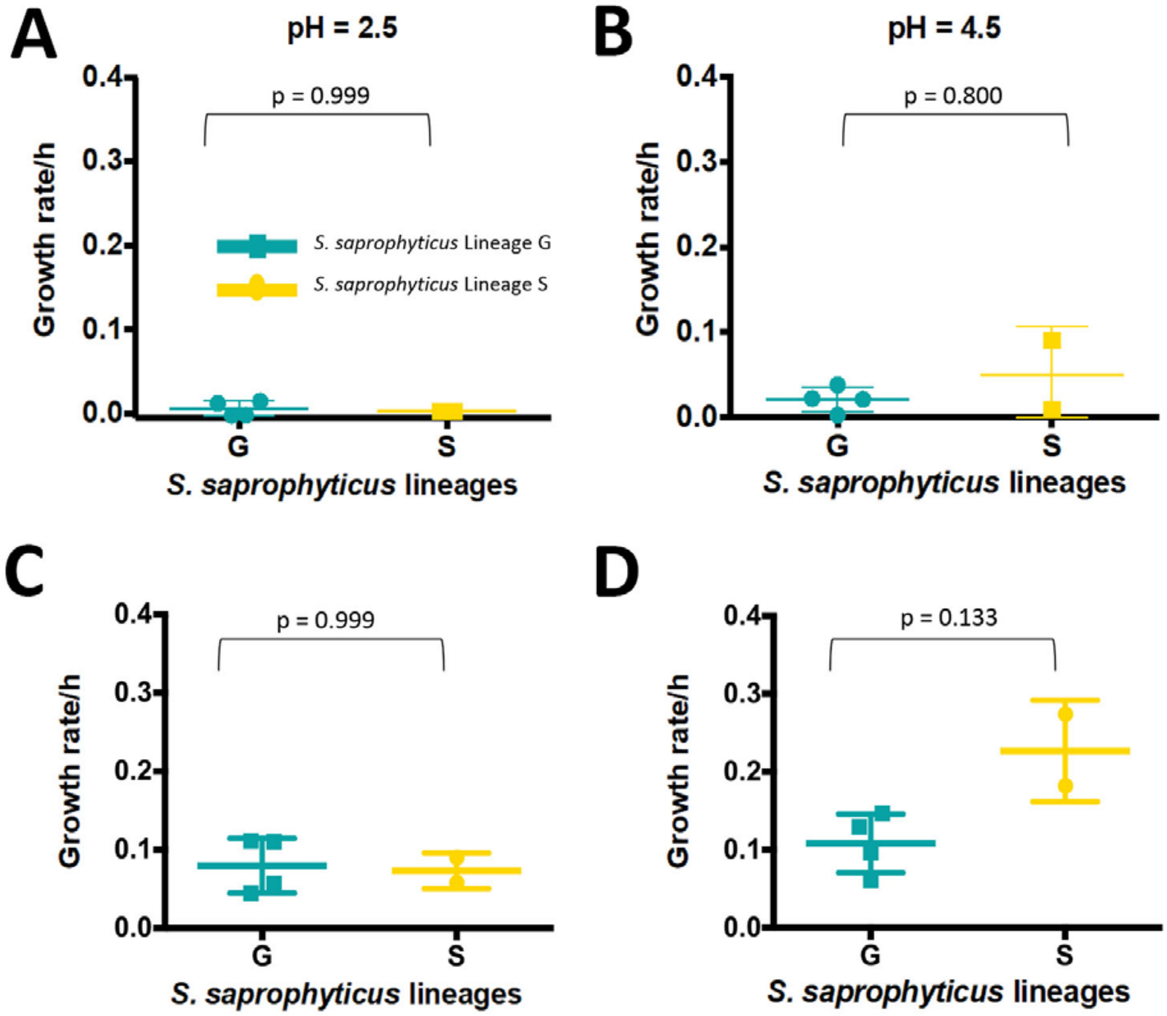

Appendix 2 Figure 4. Growth rate of *Staphylococcus saprophyticus* clonal lineages in different pH levels. A) pH 2.5 representing pH of the human stomach; B) pH 4.5 and C) pH 5.5 representing pH of human skin; and D) pH 8.0 representing pH of urine from a healthy human. Isolates were completely inhibited at pH 2.5 but grew at a low rate when pH = 4.5 and 5.5. The 2 lineages behaved slightly differently in pH = 8.0 but this difference was not statistically significant. Assays were performed in triplicates and each experiment was repeated 3 times. Error bars indicate 95% CI; horizontal lines indicate median.
